# Allergic rhinitis and asthma: inflammation in a one-airway condition

**DOI:** 10.1186/1471-2466-6-S1-S5

**Published:** 2006-11-30

**Authors:** Peter K Jeffery, Tari Haahtela

**Affiliations:** 1 Lung Pathology, Imperial College at the Royal Brompton Hospital, London, SW3 6NP, UK; 2 Skin and Allergy Hospital, Helsinki University Central Hospital, PO Box 160, 00029 HUS, Finland

## Abstract

**Background:**

Allergic rhinitis and asthma are conditions of airway inflammation that often coexist.

**Discussion:**

In susceptible individuals, exposure of the nose and lungs to allergen elicits early phase and late phase responses. Contact with antigen by mast cells results in their degranulation, the release of selected mediators, and the subsequent recruitment of other inflammatory cell phenotypes. Additional proinflammatory mediators are released, including histamine, prostaglandins, cysteinyl leukotrienes, proteases, and a variety of cytokines, chemokines, and growth factors. Nasal biopsies in allergic rhinitis demonstrate accumulations of mast cells, eosinophils, and basophils in the epithelium and accumulations of eosinophils in the deeper subepithelium (that is, lamina propria). Examination of bronchial tissue, even in mild asthma, shows lymphocytic inflammation enriched by eosinophils. In severe asthma, the predominant pattern of inflammation changes, with increases in the numbers of neutrophils and, in many, an extension of the changes to involve smaller airways (that is, bronchioli). Structural alterations (that is, remodeling) of bronchi in mild asthma include epithelial fragility and thickening of its reticular basement membrane. With increasing severity of asthma there may be increases in airway smooth muscle mass, vascularity, interstitial collagen, and mucus-secreting glands. Remodeling in the nose is less extensive than that of the lower airways, but the epithelial reticular basement membrane may be slightly but significantly thickened.

**Conclusion:**

Inflammation is a key feature of both allergic rhinitis and asthma. There are therefore potential benefits for application of anti-inflammatory strategies that target both these anatomic sites.

## Introduction

Asthma is a chronic, inflammatory condition of the lower airways characterized by largely reversible airflow obstruction, airway hyperresponsiveness, and episodic respiratory symptoms, including wheezing, productive cough, and the sensations of breathlessness and chest tightness [1]. Allergic rhinitis (AR), also often associated with conjunctival symptoms, is a disorder of the upper airways (namely, above the larynx) resulting from IgE-mediated inflammation of the nose upon contact of the nasal mucosa with allergens: symptoms include rhinorrhea, nasal itching, sneezing, and nasal obstruction [2]. The patterns of inflammation, when stable or in response to experimental allergen challenge, are similar in the upper and lower airways. Moreover, both asthma and AR may be associated with evidence of systemic inflammation. Often, as discussed in the prior article in this supplement [3], asthma and AR are comorbid conditions [2,4], with AR being a major risk factor for the occurrence of asthma [5].

In the present article, we summarize the patterns of inflammation of the upper and lower airways; we emphasize the need to consider treating the inflammation that is common to both. We introduce briefly techniques currently used to describe characteristic cells and mediators of inflammation common to both asthma and AR. Of course, patients with asthma, as well as AR, can demonstrate a spectrum of symptoms clinically and, likewise, of inflammatory changes. As more data emerge it is likely that distinct phenotypes of both AR and asthma will become accepted. Despite this, there are some obvious similarities in the patterns of inflammation yet differences in the extent of remodeling.

## Evaluating the airways for inflammation

With the exception of the anterior nares and a good proportion of the nasopharynx and larynx (which are keratinized and stratified squamous, respectively), the microscopic anatomy of normal nasal and bronchial mucosa is similar: a pseudostratified epithelium resting on a reticular basement membrane with underlying subepithelium (that is, lamina propria). The epithelium is composed of columnar ciliated cells interspersed by goblet cells [6], and beneath (in the subepithelium and submucosa) there are blood vessels, fibroblasts, nerves, mucous glands, and immune cells. The differences between nose and bronchi include the greater abundance of subepithelial capillaries and the venous cavernous sinusoids present in the nose and the presence of encircling airway smooth muscle in the lower airways, absent from the nose [6,7].

There are several techniques for evaluating inflammation in lower or upper airways in both clinical and research settings. The lower airways can be investigated by rigid or flexible fiberoptic rhinoscopy and bronchoscopy [8-10]. Bronchoscopy is used to collect endobronchial biopsies or brushings as well as bronchoalveolar lavage. Nasal sampling includes biopsy, with and without the aid of rhinoscopy, and cytology can be assessed in lavage or nasal swabs [11]. Expelled secretions for analysis include induced sputum from the lower airways and nasal mucus from the upper airways. Biopsy permits histopathological, immunohistological, and molecular examination of respiratory mucosa. Cytological examination of secretions can add useful information to that obtained by direct examination of the mucosa: these complementary techniques sample two distinct compartments [12]. Measurement of biological markers in airway and nasal secretions can also be made before and after endobronchial provocation with allergen or pharmacological or physical agents in order to study the dynamics of the inflammatory responses to such challenges [9,13].

Bronchoscopy has its limitations. It samples only a small portion of the entire lung and in its airways, only the superficial portion of the bronchial wall is sampled. While possible, examination of the smaller (2–3 mm) airways is difficult in practice and there are greater safety and ethical issues. Moreover, bronchoscopy may not be practical in severely compromised adults or pediatric patients [9] although, in the latter, recent studies have been published that aim to elucidate the patterns of inflammation and remodeling that occur as asthma becomes first recognized clinically [14-16]. The usefulness of nasal and bronchial cytology is also limited by considerable intra-individual variability as well as variability according to collection technique [11,13,17]. In disorders of airflow obstruction, there is now a greater appreciation of the need to also understand the distinct contributions to variability in the lower airways [18]. Additionally, the fractional concentration of exhaled nitric oxide may be a useful measure to assess airway inflammation in diagnostic work or to monitor the effects of anti-inflammatory therapy, even in children [19-23]. Finally, peripheral blood eosinophilia is common in AR and asthma, and it reflects the presence of systemic inflammation [24].

## Changes in the upper airways in allergic rhinitis

The inflammatory cascade in the nose begins with allergen deposition on the nasal mucosa and consists of early phase responses and, in many cases, late phase responses in susceptible individuals. Upon activation by antigen via cross-linking of IgE receptors, sensitized mast cells immediately degranulate, releasing a variety of inflammatory mediators, including histamine, prostaglandin D_2_, cysteinyl leukotrienes, and neutral proteases [2,25]. These mediators cause sensory neural stimulation and plasma exudation from blood vessels, which the patient experiences as itching, sneezing, nasal discharge, and congestion. Other localized cells involved in the allergic response include Langerhans' cells, which are dendritic cells that present antigen to the mast cells, and T helper type 2 lymphocytes, which indirectly regulate the production of IgE. Recruitment of inflammatory cells, including eosinophils, basophils, and T cells, results in further release of histamine and leukotrienes, as well as other compounds including proinflammatory cytokines and chemokines, sustaining the allergic response and promoting the late phase response that may occur 6–9 hours after allergen exposure [26,27].

Nasal biopsies of patients with active AR show accumulations of mast cells, eosinophils, and basophils in the epithelium and an accumulation of eosinophils in the deeper lamina propria [27]. In time, the reticular basement membrane may appear slightly but significantly thickened, but not to the extent seen in the lower airways in asthma (see discussion of remodeling below). Remodeling in the nose appears to be less extensive than that in the lower airways [6,24]. While this requires further study, two reasons postulated for the differences in remodeling between upper and lower airways include the secretory activity of smooth muscle cells present in bronchi but not in the nose, and the differences in embryologic origins of the bronchi and the nose [6].

## Changes in the lower airways in asthma: inflammation and remodeling

Both inflammation and structural changes (referred to as remodeling) occur in the tracheobronchial tree of patients with asthma. It has been generally considered that chronic eosinophilic inflammation is a prerequisite for the development of remodeling, and there is some animal experimental evidence to support this [28]. Investigations in humans to discover whether chronic inflammation leads to remodeling or whether remodeling begins first are in their infancy [14-16,29]. Such studies are required so that we may determine if and when there may be a 'window of opportunity' for prevention of the structural and inflammatory changes associated with the asthma phenotype. Moreover, it may become possible to predict which preschool 'wheezers' will go on to develop asthma.

Airway inflammation can be present even in patients with normal lung function but with symptoms indicative of asthma [30]; hence, measurements of the forced expiratory volume in 1 second do not reflect well or predict airway inflammation. Conversely, symptomatic infants with reversible airflow obstruction (that is, asthma) may not demonstrate evidence of either bronchial tissue eosinophilia or remodeling [15]. Considering that these pathological changes are present and maximal in severely asthmatic school children of median age 10 years [14], the changes must begin earlier. There are now new data indicating that the changes begin between the ages of 1 and 3 years and that there is a positive relationship between tissue eosinophilia and reticular basement membrane thickening at this time [29].

**Figure 1:**
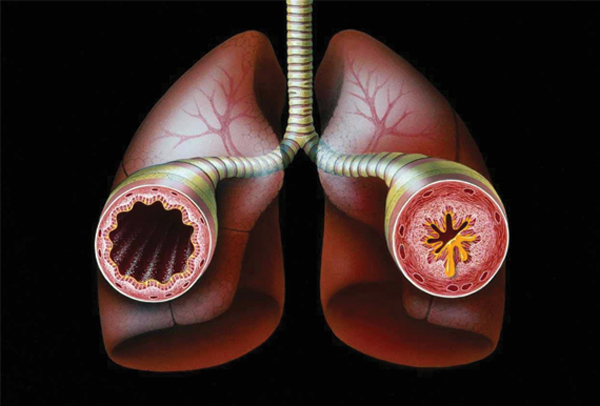
**Thickening of the bronchial wall in asthma**. Schematic illustrating the thickening of the bronchial wall in asthmatic conditions (right) as compared with normal conditions (left). Reprinted with permission from Jeffery [65].

**Figure 2:**
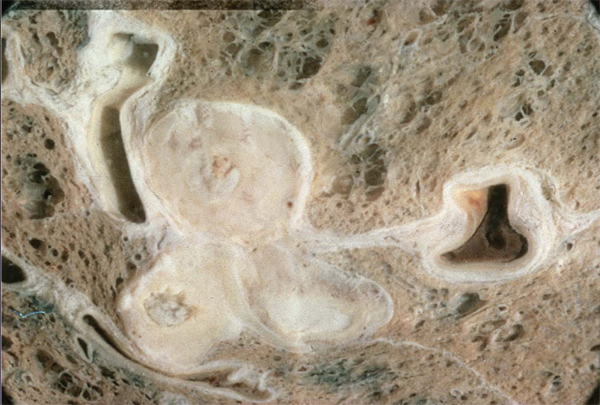
**Plugging of the airways in fatal asthma**. Gross view of a sliced lung specimen to show plugging of the airways in fatal asthma. Reprinted with permission from Jeffery [65].

Chronic inflammation plus remodeling contribute to airway wall thickening, which encroaches upon the airway lumen and increases resistance to airflow (Figure 1). Airway secretions also contribute to the pathology of asthma, and especially to the consequences of acute, severe, life-threatening exacerbations. In those rare cases when a fatal asthma attack has occurred after a short duration of asthma, airway wall thickening may not be present – and in these cases patient demise is assumed to be secondary to asphyxiation from tenacious, sticky mucus admixed with an inflammatory eosinophilic exudate in the airways (Figure 2) [31].

### Inflammation in asthma

**Figure 3:**
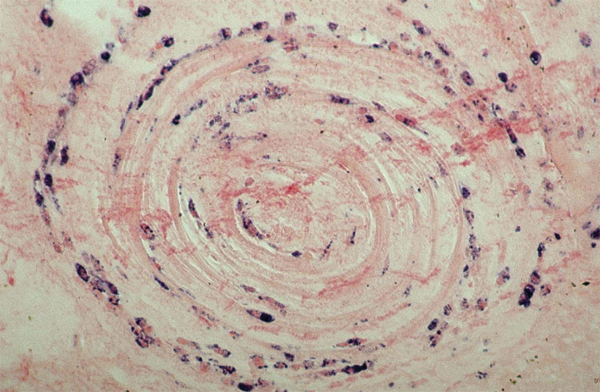
**Plug of eosinophil-rich inflammatory exudate and mucus in the airway of a patient with asthma**. Concentric lamellae of eosinophils appear blue (alkaline phosphatase antialkaline phosphatase). Reprinted with permission from Jeffery [65].

**Figure 4:**
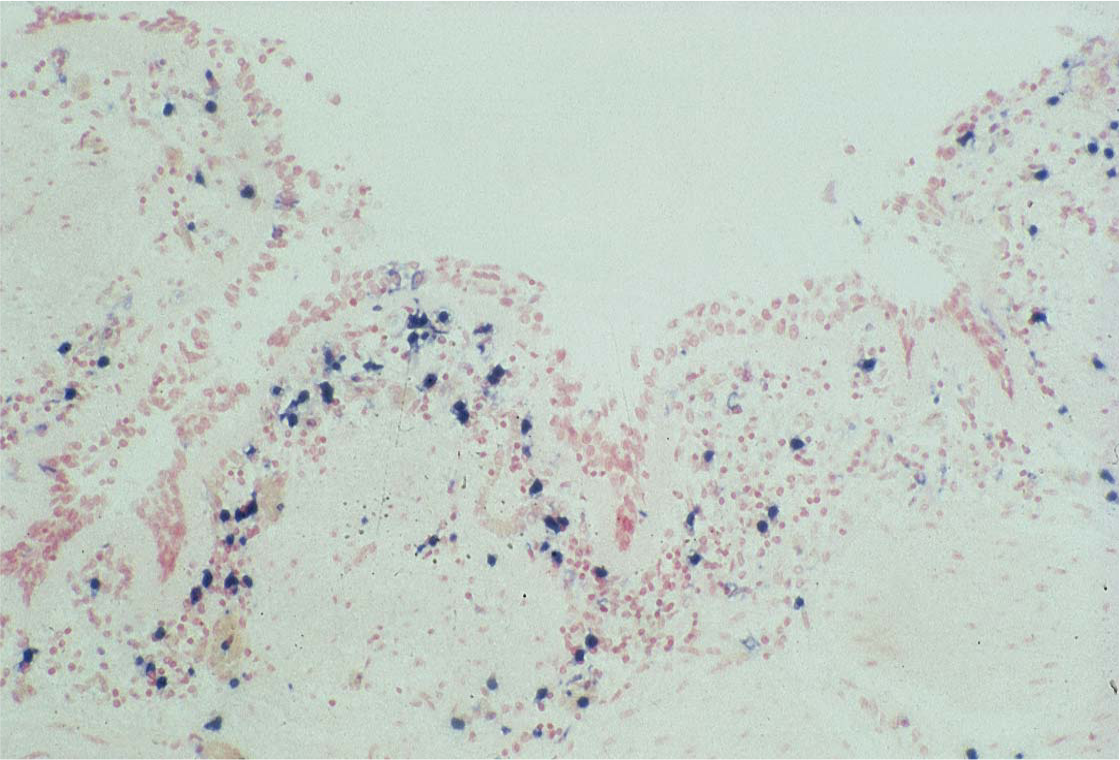
**Eosinophils in the airway wall in fatal asthma**. Fatal asthma with eosinophils (blue) in the airway wall showing the origin of the eosinophils that migrate across the mucosa, enter the lumen, and contribute to the inflammatory exudate shown in the previous figure. Reprinted with permission from Jeffery [65].

Lymphocytic inflammation enriched by eosinophils is characteristic of the bronchi in mild asthma in both adults and school children, and also of the bronchi and luminal secretions in fatal asthma (Figures 3 and 4). In severe disease the predominant pattern changes because, in addition to eosinophils, neutrophils increase, and the inflammation may spread to include the small airways (that is, airways 2 mm or smaller in diameter) [32].

In allergic asthma, inhaled allergens that penetrate the mucociliary lining layer enter the airway epithelium either via the tight junctions that surround the apical zone of bronchial epithelial cells or by direct uptake by the cells *per se*. As occurs in the nose, the sequence of reactions in the lungs may include both early phase and late phase responses. There is presentation of antigen to mast cells, cross-linking of cell surface IgE, degranulation of mast cells, release of mediators such as histamine and leukotrienes that markedly increase vascular permeability, followed by recruitment of more inflammatory cells, and further release of proinflammatory mediators.

**Figure 5:**
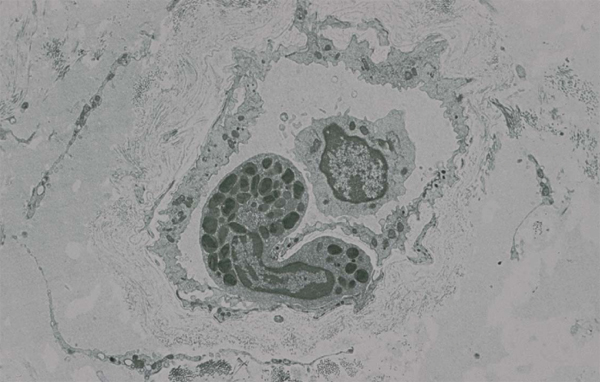
**Eosinophil emerging from a bronchial vessel to enter the bronchial mucosa**. Eosinophil emerging from a bronchial vessel to enter bronchial mucosa, as seen by transmission electron microscopy. Reprinted with permission from Jeffery [65].

In asthma, the predominant orchestrator of the chronic inflammation is the CD4 or T-helper lymphocyte, producing key regulatory cytokines such as IL-5 and IL-4 [33]. Eosinophils (originating in the bone marrow) are released into the circulation, resulting in blood eosinophilia, partly in response to IL-5. The eosinophils are selectively retained at bronchial microvascular surfaces by tumor necrosis factor alpha-induced and IL-4-induced upregulation of adhesive molecules. Once retained they are recruited into the mucosa (Figure 5) and migrate to the surface epithelium where they cross it, in response to eosinophil chemoattractants released by structural and immune elements. The then-activated eosinophils release highly toxic granules that damage the surface epithelial cells, loosening their attachments and resulting in their loss into the airway lumen, where they admix with both eosinophils and excess mucin.

**Figure 6:**
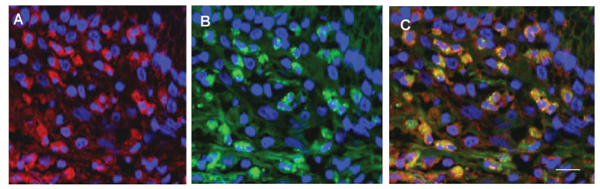
**Colocalization of the cysteinyl leukotriene type 1 receptor with eosinophils in asthma exacerbation**. Double immunofluorescence staining for identification of colocalization of the cysteinyl leukotriene type1 (cysLT_1_) receptor with eosinophils in a bronchial biopsy from a patient with asthma with severe exacerbation. (a) cysLT_1_-receptor protein immunopositivity is illustrated with Texas red fluorescence. (b) Eosinophils stained with anti-human EG_2 _coupled to fluorescein isothiocyanate conjugate. (c) EG_2_^+ ^eosinophils (internal scale bar = 10 μm). Nuclei are counterstained blue with 4',6-diamidino-2-phenylindole. EG_2 _is a monoclonal antibody to the cleaved (activated) form of eosinophil cationic protein. Reprinted with permission from Zhu and colleagues [35].

**Figure 7:**
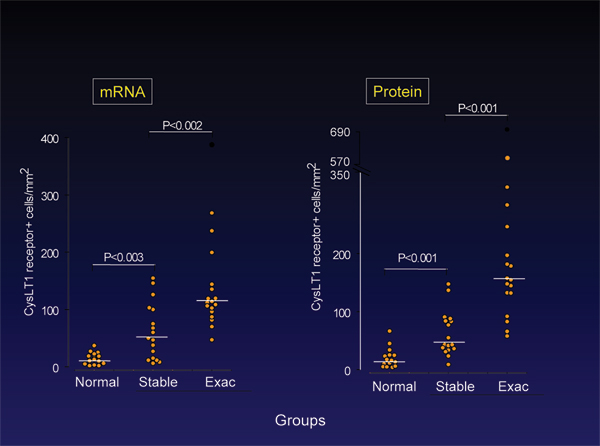
**Cysteinyl leukotriene type 1 receptor mRNA and protein-positive cells in bronchial biopsies**. Graphs of counts for cysteinyl leukotriene type 1 (cysLT_1_) receptor mRNA and protein-positive cells in bronchial biopsies of nonsmoker control (normal), stable asthma, and exacerbated asthma (exac) groups. Data expressed as the number of positive cells per mm^2 ^of subepithelium. Dots show individual counts and horizontal bars show median values (Mann-Whitney U test). Reprinted with permission from Zhu and colleagues [35].

Eosinophil chemoattractants include, for example, eotaxin, macrophage/monocyte chemotactic protein 4, RANTES, and cysteinyl leukotrienes [34]; these chemoattractants act on distinct cell surface receptors (CC chemokine receptor 3 and cysteinyl leukotriene type 1 (cysLT_1_) receptor) present on the eosinophil, but not exclusively so. In support of this, challenge with leukotriene E_4 _results in greatly increased numbers of eosinophils in the bronchial wall [34]. Moreover, a recent study in asthma has used the molecular techniques of *in situ *hybridization and immunohistochemistry to localize cells that express either the mRNA or the protein for the cysLT_1 _receptor, respectively [35]. In addition to the eosinophil, the receptor appears to be present on a variety of bronchial inflammatory cells (Figure 6), including neutrophils, mast cells, macrophages, B lymphocytes, and plasma cells. The study also demonstrated that the numbers of inflammatory cells expressing the cysLT_1 _receptor are increased – in comparison with normal healthy nonsmokers – in nonsmoking patients with mild, stable asthma and that there is a further increase among patients experiencing a severe exacerbation of asthma leading to hospitalization (Figure 7) [35].

### Remodeling in asthma

Remodeling in asthma has been defined as a change in structure that is inappropriate to the maintenance of normal airway function [33]. Remodeling is evident even in newly diagnosed or mild asthma and is characterized by epithelial fragility and reticular basement membrane thickening. With increasing severity of asthma, there are increases of airway smooth muscle mass, vascularity, numbers of fibroblasts, and interstitial collagen, as well as mucous gland hypertrophy [33]. These changes appear to be greatest in the larger, more proximal airways.

**Figure 8:**
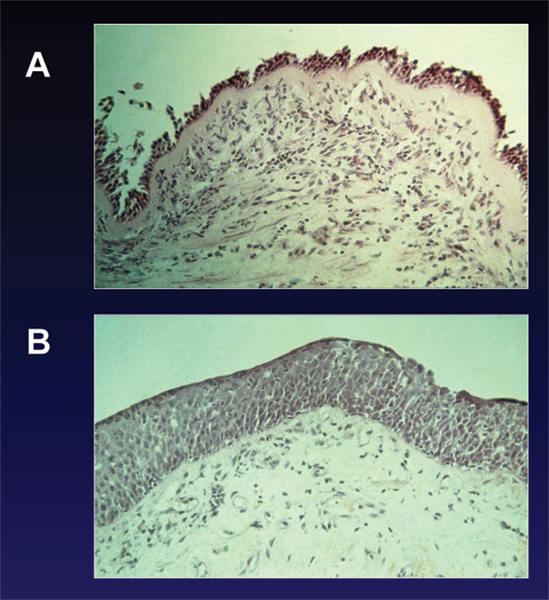
**Bronchial biopsies in asthma and chronic obstructive pulmonary disease**. (a) Bronchial biopsy in a nonsmoker with mild asthma. Note thickening of the epithelial reticular basement membrane in comparison with (b). (b) Bronchial biopsy in a smoker with chronic obstructive pulmonary disease (alkaline phosphatase antialkaline phosphatase, original ×240). Reprinted with permission from Jeffery [66].

Thickening of the reticular basement membrane occurs early in asthma (Figure 8), even before diagnosis, and is detected in children with mild asthma [16]. It most probably reflects the response to ongoing epithelial injury and regeneration, in which eosinophils play a role [36]. In school children between the ages of 6 and 16 with severe asthma the membrane is already maximally thickened, but there is no significant association between its thickness and age or symptom duration [14]. These changes appear in preschool wheezy children by the age of 29 months [29].

Airway smooth muscle surrounds the airways as two opposing helices; namely, in a geodesic pattern. As the muscle shortens, therefore, it not only constricts but tends to shorten the airway against an elastic load. Leukotrienes are very powerful constrictors of airway smooth muscle, being 1000-fold or 2000-fold more active than histamine. There are at least three possible mechanisms of smooth muscle mass enlargement in asthma: myocyte hypertrophy, myocyte hyperplasia due to cell division and proliferation, or myocyte de-differentiation and migration across the mucosa in the form of myofibroblasts or fibromyocytes. It is speculated that these may then re-differentiate to form new blocks of smooth muscle that come to lie just below (external to) the epithelium [33].

Greater numbers of mast cells have recently been found located within the bronchial smooth muscle of patients with asthma than in those with eosinophilic bronchitis – the latter is also a condition of airway eosinophilia and remodeling but without the functional abnormalities characteristic of asthma. The difference in smooth muscle mast cell number that discriminates between these conditions has led to the hypothesis that the infiltration of airway smooth muscle by mast cells is responsible for the disordered airway function characteristic of asthma, and thus that asthma is the result of a mast cell myositis [37]. These and other data highlight the importance of the localization of inflammatory cells to distinct tissue compartments rather than their overall number *per se*. No doubt there will be many more studies to test this hypothesis.

## Interactions between asthma and allergic rhinitis

Allergic asthma and AR are often considered clinical manifestations of the same condition, the chronic allergic respiratory syndrome [38,39]. The many epidemiological associations between the two conditions are reviewed in the prior article in this supplement [3]. Moreover, as discussed previously [3], bronchial hyperresponsiveness is common in people with AR, even if they have no symptoms of asthma, and bronchial inflammation can result from nasal allergen challenge in patients with AR in the absence of obvious asthma [40]. Conversely, patients with asthma can have eosinophilic infiltration of their nasal mucosa without reporting the symptoms of rhinitis [38,39]. Segmental bronchial provocation in patients with AR but not asthma has been shown to induce nasal inflammation [41,42]. Not all patients with asthma have rhinitis, however, and not all patients with rhinitis have asthma. Genetic differences contribute to this discrepancy; for example, certain haplotypes of the newly identified GPR154 gene on chromosome 7 predispose individuals to IgE-mediated rhinitis but not to asthma [43].

Possible mechanisms for the influence of AR on lower airways include disturbance of the beneficial role of nasal mucosa in conditioning the air entering the respiratory tree; neural interaction between upper and lower airways; irritant effects of nasal secretions directly entering the lower airways; and systemic propagation of nasal inflammation to the bronchial mucosa (or vice versa) via effects of mediators and inflammatory cells on bone marrow – 'systemic cross-talk' [38,40,44].

## Effects of anti-inflammatory treatment in allergic rhinitis and asthma – proof of concept

Nasal corticosteroids, antihistamines, and the leukotriene receptor antagonist (LTRA) montelukast have each been shown to have anti-inflammatory effects on different aspects of inflammation in AR, and these effects have been recently reviewed [45].

In patients with asthma, inhalation of corticosteroids (ICS) produces reductions in bronchial inflammation by days or weeks, whereas reductions in reticular basement membrane thickening are achieved over longer periods, after about 1 year [46]. Treatment with montelukast reduces peripheral eosinophil counts and eosinophils in induced sputum [47]. Short-term treatment (that is, 4 weeks) with another LTRA, pranlukast, reduces bronchial inflammation in endobronchial biopsies [48]. Moreover, there is *in vitro *evidence from isolates and cultures of human airway smooth muscle that leukotrienes enhance myocyte proliferation and migration, processes that can be attenuated by LTRAs [49-51].

The clinical benefits of early treatment with anti-inflammatory therapy for asthma were shown in studies in the early 1990s [52,53]. In 1994, the Finnish asthma program was established to reduce morbidity of asthma in Finland with a focus on treating inflammation, as implemented by a network of asthma-responsible doctors, nurses, and pharmacists. While this program has not halted the increase of asthma in Finland, disability pensions and the number of hospital bed-days for asthma and deaths from asthma have considerably decreased over 10 years [54]. This outcome can be attributed mainly to more effective and early use of anti-inflammatory medication.

Several recent studies have shown that there is still room for improvement in anti-inflammatory treatment of patients with asthma. There are several arguments that favor the combined use of ICS and an LTRA in asthma. First, cysteinyl leukotriene levels in induced sputum remain increased in patients with asthma receiving ICS, suggesting that corticosteroids do not suppress leukotriene production [55]. Also, the addition of the LTRA montelukast improves clinical endpoints, with a 56% increase in asthma-free days, for patients with persistent disease who were receiving ICS [56]. Finally, in the IMPACT study, montelukast added to fluticasone gave the same level of protection against asthma exacerbation as did the addition of a long-acting β_2_-agonist [57]. Among the Finnish patients in this study, the mean sputum eosinophil count fell significantly at week 24 in those receiving montelukast but not in those patients receiving salmeterol [57].

For patients with comorbid AR and asthma, effective management of their rhinitis may also improve the coexisting asthma [2]. As discussed in the preceding paper of the present supplement [3], observational studies have shown the benefits of treating rhinitis in terms of reduced risk of hospitalizations or emergency department visits for asthma [58]. Montelukast improves lung function in patients who have rhinitis [59,60], and daily rhinitis symptoms improve when montelukast is given to patients with hay fever and asthma – as do patients' and physicians' global evaluations of asthma symptoms [61].

Finally, the World Allergy Organization, in conjunction with the World Health Organization, has recently published Guidelines for the Prevention of Allergy and Allergic Asthma that include primary, secondary, and tertiary prevention strategies [62,63]. The guidelines recommend treating upper airway disease, such as AR, to prevent the development of asthma. Evidence to show any preventive effect of this strategy, however, is mostly lacking. There are indications that pollen immunotherapy reduces the development of asthma in children with seasonal rhinoconjunctivitis [64], but again effects on so-called allergic march are not known. Tertiary prevention strategies in the World Allergy Organization/World Health Organization guidelines – to prevent exacerbations and disease progression – emphasize the need to treat the underlying inflammatory process.

## Conclusion

As inflammation and its relationship to remodeling in AR and asthma are becoming better understood, the importance of anti-inflammatory treatment is increasingly accepted. For patients with symptoms of asthma, it is important to detect inflammation (and remodeling) early and to control inflammation in all its stages and severities. Initial therapies comprise anti-inflammatory medication, usually ICS or LTRA in mild asthma or ICS in more severe asthma. This is usually supplemented with rapidly acting β_2_-agonist as needed. In moderate to severe asthma, regular long-acting β_2_-agonists are used if anti-inflammatory therapy fails to control the disease, as indicated by increasing daily requirement for short-acting β_2_-agonist. The combination of ICS and oral LTRA anti-inflammatory therapy is one alternative approach to treatment and provides the option of treating airway inflammation as a whole. Given the similarity that exists between the patterns of inflammation seen in AR and asthma, patients may best benefit from an approach that considers treating the entire airway rather than only a part, as well as the skin as necessary.

## Abbreviations

AR = allergic rhinitis; cysLT_1 _= cysteinyl leukotriene type 1; ICS = inhalation of corticosteroids; IL = interleukin; LTRA = leukotriene receptor antagonist; RANTES = regulated upon activation, normal T-lymphocyte expressed.

## Competing interests

PKJ has received funds to support independently initiated research and lecture fees from Merck & Co, USA. TH has received speaker's fees from Merck & Co.

## References

[B1] Bousquet J, Jeffery PK, Busse WW, Johnson M, Vignola AM (2000). Asthma. From bronchoconstriction to airways inflammation and remodeling. Am J Respir Crit Care Med.

[B2] Bousquet J, Van Cauwenberge P, Khaltaev N (2001). Allergic rhinitis and its impact on asthma. J Allergy Clin Immunol.

[B3] Thomas M (2006). Allergic rhinitis: evidence for impact on asthma. BMC Pulm Med.

[B4] Linneberg A, Henrik Nielsen N, Frolund L, Madsen F, Dirksen A, Jorgensen T (2002). The link between allergic rhinitis and allergic asthma: a prospective population-based study. The Copenhagen Allergy Study. Allergy.

[B5] Guerra S, Sherrill DL, Martinez FD, Barbee RA (2002). Rhinitis as an independent risk factor for adult-onset asthma. J Allergy Clin Immunol.

[B6] Bousquet J, Jacot W, Vignola AM, Bachert C, Van Cauwenberge P (2004). Allergic rhinitis: a disease remodeling the upper airways?. J Allergy Clin Immunol.

[B7] Jeffery PK, Busse W, Holgate ST (1995). Structural, immunologic, and neural elements of the normal human airway wall. Asthma and Rhinitis.

[B8] Sullivan WB, Linehan ATt, Hilman BC, Walcott DW, Nandy I (1996). Flexible fiberoptic rhinoscopy in the diagnosis of nasal polyps in cystic fibrosis. Allergy Asthma Proc.

[B9] Busse WW, Wanner A, Adams K, Reynolds HY, Castro M, Chowdhury B, Kraft M, Levine RJ, Peters SP, Sullivan EJ (2005). Investigative bronchoprovocation and bronchoscopy in airway diseases. Am J Respir Crit Care Med.

[B10] Jeffery P, Holgate S, Wenzel S (2003). Methods for the assessment of endobronchial biopsies in clinical research: application to studies of pathogenesis and the effects of treatment. Am J Respir Crit Care Med.

[B11] Deutschle T, Friemel E, Starnecker K, Riechelmann H (2005). Nasal cytologies – impact of sampling method, repeated sampling and interobserver variability. Rhinology.

[B12] Lim MC, Taylor RM, Naclerio RM (1995). The histology of allergic rhinitis and its comparison to cellular changes in nasal lavage. Am J Respir Crit Care Med.

[B13] Riechelmann H, Deutschle T, Friemel E, Gross HJ, Bachem M (2003). Biological markers in nasal secretions. Eur Respir J.

[B14] Payne DN, Rogers AV, Adelroth E, Bandi V, Guntupalli KK, Bush A, Jeffery PK (2003). Early thickening of the reticular basement membrane in children with difficult asthma. Am J Respir Crit Care Med.

[B15] Saglani S, Malmstrom K, Pelkonen AS, Malmberg LP, Lindahl H, Kajosaari M, Turpeinen M, Rogers AV, Payne DN, Bush A (2005). Airway remodeling and inflammation in symptomatic infants with reversible airflow obstruction. Am J Respir Crit Care Med.

[B16] Barbato A, Turato G, Baraldo S, Bazzan E, Calabrese F, Tura M, Zuin R, Beghe B, Maestrelli P, Fabbri LM (2003). Airway inflammation in childhood asthma. Am J Respir Crit Care Med.

[B17] Belda J, Parameswaran K, Keith PK, Hargreave FE (2001). Repeatability and validity of cell and fluid-phase measurements in nasal fluid: a comparison of two methods of nasal lavage. Clin Exp Allergy.

[B18] Gamble E, Qiu Y, Wang D, Zhu J, Vignola AM, Kroegel C, Morell F, Hansel TT, Pavord ID, Rabe KF (2006). Variability of bronchial inflammation in chronic obstructive pulmonary disease: implications for study design. Eur Respir J.

[B19] Spallarossa D, Battistini E, Silvestri M, Sabatini F, Fregonese L, Brazzola G, Rossi GA (2003). Steroid-naïve adolescents with mild intermittent allergic asthma have airway hyperresponsiveness and elevated exhaled nitric oxide levels. J Asthma.

[B20] Bisgaard H, Loland L, Oj JA (1999). NO in exhaled air of asthmatic children is reduced by the leukotriene receptor antagonist montelukast. Am J Respir Crit Care Med.

[B21] Buchvald F, Baraldi E, Carraro S, Gaston B, De Jongste J, Pijnenburg MW, Silkoff PE, Bisgaard H (2005). Measurements of exhaled nitric oxide in healthy subjects age 4 to 17 years. J Allergy Clin Immunol.

[B22] Buchvald F, Eiberg H, Bisgaard H (2003). Heterogeneity of FeNO response to inhaled steroid in asthmatic children. Clin Exp Allergy.

[B23] Payne DN, Adcock IM, Wilson NM, Oates T, Scallan M, Bush A (2001). Relationship between exhaled nitric oxide and mucosal eosinophilic inflammation in children with difficult asthma, after treatment with oral prednisolone. Am J Respir Crit Care Med.

[B24] Braunstahl GJ, Fokkens WJ, Overbeek SE, KleinJan A, Hoogsteden HC, Prins JB (2003). Mucosal and systemic inflammatory changes in allergic rhinitis and asthma: a comparison between upper and lower airways. Clin Exp Allergy.

[B25] Naclerio RM (1991). Allergic rhinitis. N Engl J Med.

[B26] Kay AB (2001). Allergy and allergic diseases. First of two parts. N Engl J Med.

[B27] Howarth PH, Salagean M, Dokic D (2000). Allergic rhinitis: not purely a histamine-related disease. Allergy.

[B28] Humbles AA, Lloyd CM, McMillan SJ, Friend DS, Xanthou G, McKenna EE, Ghiran S, Gerard NP, Yu C, Orkin SH (2004). A critical role for eosinophils in allergic airways remodeling. Science.

[B29] Saglani S, Payne DN, Nicholson AG, Jeffery PK, Bush A (2005). Thickening of the epithelial reticular basement membrane in pre-school children with troublesome wheeze [abstract]. American Thoracic Society International Conference; 2005; San Diego, CA.

[B30] Rytila P, Metso T, Heikkinen K, Saarelainen P, Helenius IJ, Haahtela T (2000). Airway inflammation in patients with symptoms suggesting asthma but with normal lung function. Eur Respir J.

[B31] Bai TR, Cooper J, Koelmeyer T, Pare PD, Weir TD (2000). The effect of age and duration of disease on airway structure in fatal asthma. Am J Respir Crit Care Med.

[B32] Balzar S, Wenzel SE, Chu HW (2002). Transbronchial biopsy as a tool to evaluate small airways in asthma. Eur Respir J.

[B33] Jeffery PK (2004). Remodeling and inflammation of bronchi in asthma and chronic obstructive pulmonary disease. Proc Am Thorac Soc.

[B34] Laitinen LA, Laitinen A, Haahtela T, Vilkka V, Spur BW, Lee TH (1993). Leukotriene E4 and granulocytic infiltration into asthmatic airways. Lancet.

[B35] Zhu J, Qiu YS, Figueroa DJ, Bandi V, Galczenski H, Hamada K, Guntupalli KK, Evans JF, Jeffery PK (2005). Localization and upregulation of cysteinyl leukotriene-1 receptor in asthmatic bronchial mucosa. Am J Respir Cell Mol Biol.

[B36] Knight DA, Holgate ST (2003). The airway epithelium: structural and functional properties in health and disease. Respirology.

[B37] Brightling CE, Bradding P, Symon FA, Holgate ST, Wardlaw AJ, Pavord ID (2002). Mast-cell infiltration of airway smooth muscle in asthma. N Engl J Med.

[B38] Togias A (2003). Rhinitis and asthma: evidence for respiratory system integration. J Allergy Clin Immunol.

[B39] Gaga M, Lambrou P, Papageorgiou N, Koulouris NG, Kosmas E, Fragakis S, Sofios C, Rasidakis A, Jordanoglou J (2000). Eosinophils are a feature of upper and lower airway pathology in non-atopic asthma, irrespective of the presence of rhinitis. Clin Exp Allergy.

[B40] Bonay M, Neukirch C, Grandsaigne M, Lecon-Malas V, Ravaud P, Dehoux M, Aubier M (2006). Changes in airway inflammation following nasal allergic challenge in patients with seasonal rhinitis. Allergy.

[B41] Braunstahl GJ, Kleinjan A, Overbeek SE, Prins JB, Hoogsteden HC, Fokkens WJ (2000). Segmental bronchial provocation induces nasal inflammation in allergic rhinitis patients. Am J Respir Crit Care Med.

[B42] Braunstahl GJ, Overbeek SE, Fokkens WJ, Kleinjan A, McEuen AR, Walls AF, Hoogsteden HC, Prins JB (2001). Segmental bronchoprovocation in allergic rhinitis patients affects mast cell and basophil numbers in nasal and bronchial mucosa. Am J Respir Crit Care Med.

[B43] Melen E, Bruce S, Doekes G, Kabesch M, Laitinen T, Lauener R, Lindgren CM, Riedler J, Scheynius A, van Hage-Hamsten M (2005). Haplotypes of G protein-coupled receptor 154 are associated with childhood allergy and asthma. Am J Respir Crit Care Med.

[B44] Togias A (2004). Systemic effects of local allergic disease. J Allergy Clin Immunol.

[B45] Frieri M (2005). Inflammatory issues in allergic rhinitis and asthma. Allergy Asthma Proc.

[B46] Ward C, Pais M, Bish R, Reid D, Feltis B, Johns D, Walters EH (2002). Airway inflammation, basement membrane thickening and bronchial hyperresponsiveness in asthma. Thorax.

[B47] Pizzichini E, Leff JA, Reiss TF, Hendeles L, Boulet LP, Wei LX, Efthimiadis AE, Zhang J, Hargreave FE (1999). Montelukast reduces airway eosinophilic inflammation in asthma: a randomized, controlled trial. Eur Respir J.

[B48] Nakamura Y, Hoshino M, Sim JJ, Ishii K, Hosaka K, Sakamoto T (1998). Effect of the leukotriene receptor antagonist pranlukast on cellular infiltration in the bronchial mucosa of patients with asthma. Thorax.

[B49] Jeffery PK (2001). The roles of leukotrienes and the effects of leukotriene receptor antagonists in the inflammatory response and remodelling of allergic asthma. Clin Exp Allergy Rev.

[B50] Panettieri RA, Tan EM, Ciocca V, Luttmann MA, Leonard TB, Hay DW (1998). Effects of LTD4 on human airway smooth muscle cell proliferation, matrix expression, and contraction in vitro: differential sensitivity to cysteinyl leukotriene receptor antagonists. Am J Respir Cell Mol Biol.

[B51] Parameswaran K, Cox G, Radford K, Janssen LJ, Sehmi R, O'Byrne PM (2002). Cysteinyl leukotrienes promote human airway smooth muscle migration. Am J Respir Crit Care Med.

[B52] Haahtela T, Jarvinen M, Kava T, Kiviranta K, Koskinen S, Lehtonen K, Nikander K, Persson T, Reinikainen K, Selroos O (1991). Comparison of a beta 2-agonist, terbutaline, with an inhaled corticosteroid, budesonide, in newly detected asthma. N Engl J Med.

[B53] Haahtela T, Jarvinen M, Kava T, Kiviranta K, Koskinen S, Lehtonen K, Nikander K, Persson T, Selroos O, Sovijarvi A (1994). Effects of reducing or discontinuing inhaled budesonide in patients with mild asthma. N Engl J Med.

[B54] Haahtela T, Tuomisto LE, Pietinalho A, Klaukka T, Erhola M, Kaila M, Nieminen MM, Kontula E, Laitinen LA (2006). A 10 asthma programme in Finland: major change for the better. Thorax.

[B55] Pavord ID, Ward R, Woltmann G, Wardlaw AJ, Sheller JR, Dworski R (1999). Induced sputum eicosanoid concentrations in asthma. Am J Respir Crit Care Med.

[B56] Vaquerizo MJ, Casan P, Castillo J, Perpina M, Sanchis J, Sobradillo V, Valencia A, Verea H, Viejo JL, Villasante C (2003). Effect of montelukast added to inhaled budesonide on control of mild to moderate asthma. Thorax.

[B57] Bjermer L, Bisgaard H, Bousquet J, Fabbri LM, Greening AP, Haahtela T, Holgate ST, Picado C, Menten J, Dass SB (2003). Montelukast and fluticasone compared with salmeterol and fluticasone in protecting against asthma exacerbation in adults: one year, double blind, randomised, comparative trial. BMJ.

[B58] Crystal-Peters J, Neslusan C, Crown WH, Torres A (2002). Treating allergic rhinitis in patients with comorbid asthma: the risk of asthma-related hospitalizations and emergency department visits. J Allergy Clin Immunol.

[B59] Price DB, Hernandez D, Magyar P, Fiterman J, Beeh KM, James IG, Konstantopoulos S, Rojas R, van Noord JA, Pons M (2003). Randomised controlled trial of montelukast plus inhaled budesonide versus double dose inhaled budesonide in adult patients with asthma. Thorax.

[B60] Price DB, Swern A, Tozzi CA, Philip G, Polos P (2006). Effect of montelukast on lung function in asthma patients with allergic rhinitis: analysis from the COMPACT trial. Allergy.

[B61] Philip G, Nayak AS, Berger WE, Leynadier F, Vrijens F, Dass SB, Reiss TF (2004). The effect of montelukast on rhinitis symptoms in patients with asthma and seasonal allergic rhinitis. Curr Med Res Opin.

[B62] World Health Organization (2003). Prevention of allergy and allergic asthma. Based on the WHO/WAO Meeting on the Prevention of Allergy and Allergic Asthma; 8–9 January 2002; Geneva.

[B63] Johansson SG, Haahtela T (2004). World Allergy Organization Guidelines for Prevention of Allergy and Allergic Asthma. Condensed Version. Int Arch Allergy Immunol.

[B64] Möller C, Dreborg S, Ferdousi HA, Halken S, Host A, Jacobsen L, Koivikko A, Koller DY, Niggemann B, Norberg LA (2002). Pollen immunotherapy reduces the development of asthma in children with seasonal rhinoconjunctivitis (the PAT-study). J Allergy Clin Immunol.

[B65] Jeffery PK, Adkinson FN, Yunginger J, Busse W, Bochner B, Holgate S, Simons FE (2003). Pathology of asthma compared with chronic obstructive pulmonary disease. Middleton's Allergy.

[B66] Jeffery PK (2000). Comparison of the structural and inflammatory features of COPD and asthma. Giles F. Filley Lecture. Chest.

